# Safe birth in cultural safety in southern Mexico: a pragmatic non-inferiority cluster-randomised controlled trial

**DOI:** 10.1186/s12884-021-04344-w

**Published:** 2022-01-17

**Authors:** Iván Sarmiento, Sergio Paredes-Solís, Abraham de Jesús García, Nadia Maciel Paulino, Felipe René Serrano de los Santos, José Legorreta-Soberanis, Germán Zuluaga, Anne Cockcroft, Neil Andersson

**Affiliations:** 1 CIET-Participatory Research at McGill, Faculty of Medicine and Health Sciences, Department of Family Medicine, McGill University, 5858 Chemin de la Côte des Neiges 3rd floor, Montreal, QC H3S 1Z1 Canada; 2 Grupo de Estudios en Sistemas Tradicionales de Salud, Escuela de Medicina y Ciencias de la Salud, Universidad del Rosario, Bogotá, Colombia; 3 Centro de Investigación de Enfermedades Tropicales, Universidad Autónoma de Guerrero, Acapulco, Mexico

**Keywords:** Community health worker, Traditional birth attendant, Randomised controlled trial, Equity in access, Aboriginal health, Nahua, *Na savi*/Mixteco, *Me’phaa*/Tlapaneco, *Nancue ñomndaa*/Amuzgo

## Abstract

**Background:**

Available research on the contribution of traditional midwifery to safe motherhood focuses on retraining and redefining traditional midwives, assuming cultural prominence of Western ways. Our objective was to test if supporting traditional midwives on their own terms increases cultural safety (respect of Indigenous traditions) without worsening maternal health outcomes.

**Methods:**

Pragmatic parallel-group cluster-randomised controlled non-inferiority trial in four municipalities in Guerrero State, southern Mexico, with *Nahua*, *Na savi*, *Me’phaa* and *Nancue ñomndaa* Indigenous groups. The study included all pregnant women in 80 communities and 30 traditional midwives in 40 intervention communities. Between July 2015 and April 2017, traditional midwives and their apprentices received a monthly stipend and support from a trained intercultural broker, and local official health personnel attended a workshop for improving attitudes towards traditional midwifery. Forty communities in two control municipalities continued with usual health services. Trained Indigenous female interviewers administered a baseline and follow-up household survey, interviewing all women who reported pregnancy or childbirth in all involved municipalities since January 2016. Primary outcomes included childbirth and neonatal complications, perinatal deaths, and postnatal complications, and secondary outcomes were traditional childbirth (at home, in vertical position, with traditional midwife and family), access and experience in Western healthcare, food intake, reduction of heavy work, and cost of health care.

**Results:**

Among 872 completed pregnancies, women in intervention communities had lower rates of primary outcomes (perinatal deaths or childbirth or neonatal complications) (RD -0.06 95%CI − 0.09 to − 0.02) and reported more traditional childbirths (RD 0.10 95%CI 0.02 to 0.18). Among institutional childbirths, women from intervention communities reported more traditional management of placenta (RD 0.34 95%CI 0.21 to 0.48) but also more non-traditional cold-water baths (RD 0.10 95%CI 0.02 to 0.19). Among home-based childbirths, women from intervention communities had fewer postpartum complications (RD -0.12 95%CI − 0.27 to 0.01).

**Conclusions:**

Supporting traditional midwifery increased culturally safe childbirth without worsening health outcomes. The fixed population size restricted our confidence for inference of non-inferiority for mortality outcomes. Traditional midwifery could contribute to safer birth among Indigenous communities if, instead of attempting to replace traditional practices, health authorities promoted intercultural dialogue.

**Trial registration:**

Retrospectively registered ISRCTN12397283. Trial status: concluded.

**Supplementary Information:**

The online version contains supplementary material available at 10.1186/s12884-021-04344-w.

## Plain English summary

In many Indigenous communities, traditional midwives support mothers during pregnancy, childbirth, and some days afterwards. Research involving traditional midwives has focused on training them in Western techniques and redefining their role to support Western care. In Guerrero state, Mexico, Indigenous mothers continue to trust traditional midwives. Almost half of these mothers still prefer traditional childbirths, at home, in the company of their families and following traditional practices. We worked with 30 traditional midwives to see if supporting their practice allowed traditional childbirth without worsening mothers’ health. Each traditional midwife received an inexpensive stipend, a scholarship for an apprentice and support from an intercultural broker. The official health personnel participated in a workshop to improve their attitudes towards traditional midwives. We compared 40 communities in two municipalities that received support for traditional midwifery with 40 communities in two municipalities that continued to receive usual services. We interviewed 872 women with childbirth between 2016 and 2017. Mothers in intervention communities suffered fewer complications during childbirth and had fewer complications or deaths of their babies. They had more traditional childbirths and fewer perineal tears or infections across home-based childbirths. Among those who went to Western care, mothers in intervention communities had more traditional management of the placenta but more non-traditional cold-water baths. Supporting traditional midwifery increased traditional childbirth without worsening health outcomes. The small size of participating populations limited our confidence about the size of this difference. Health authorities could promote better health outcomes if they worked with traditional midwives instead of replacing them.

**Supplementary Information:**

The online version contains supplementary material available at 10.1186/s12884-021-04344-w.

## Background

The contribution of traditional midwives to safe motherhood is the subject of ongoing debate [1–3]. Distinguishing them from Western midwives, traditional midwives have skills based on apprenticeships and “primarily on experience and knowledge acquired informally through the traditions and practices of the communities where they originated” [4]. The dominant approach of Western health services to traditional midwives treats them as unskilled birth attendants. After decades of efforts to retrain them, the current focus is to redefine the role of traditional midwives as auxiliary to or in support of conventional obstetric care [5, 6].

Several systematic reviews summarise the impact of retraining traditional midwives or involving them in intervention packages [7–12]. Low to moderate quality evidence shows improvement in “knowledge, attitude, behaviour, and advice” [11]; more referrals to the formal health system [13]; small reductions in perinatal, intrapartum and postnatal mortality [10, 11, 14–19]; statistically significant reduction in maternal morbidity [13] and, with studies of limited size, non-significant reduction in maternal mortality [10, 14, 20]. Several authors have proposed retraining of traditional midwives to do Western childbirths as a cost-effective way to reduce perinatal mortality [21, 22], with evidence less clear for reduction of maternal mortality. Our search of studies involving traditional midwives in Indigenous communities in the Americas between 1946 to 2020 (CINAHL, Scopus, LILACS, MEDLINE, Embase, Google Scholar and 15 additional web engines for grey literature) identified no published randomised controlled trial (RCT) that tested the value of traditional midwifery practices.

In response to a request from local Indigenous communities, researchers at the *Centro de Investigación de Enfermedades Tropicales* (CIET) at the *Universidad Autónoma de Guerrero* launched a participatory initiative to address poor maternal health outcomes. In this region, maternal mortality is ten times higher than in the rest of the state [23], which has the highest maternal mortality rate in Mexico [24, 25]. A 2008 survey found Indigenous women in this area preferred home childbirths over hospital childbirths, due to differences between their expectations and the available services [26]. Traditional midwives attended about one half of all childbirths, and women who gave birth attended by traditional midwives reported lower rates of perineal trauma [26]. A 2012 pilot randomised controlled trial found Indigenous women in communities where traditional midwives received support (to pay an apprentice, access a local birthing facility, and logistical assistance from a male community health worker) had reduced *birth* complications compared with their counterparts in control communities. It also suggested other contributions of the intervention, like reduced gender violence [27].

The Safe Birth in Cultural Safety trial tests whether supporting traditional midwives on their own terms results in non-inferior maternal health outcomes while increasing cultural safety around childbirth.

## Methods

This participatory research applies the principles of cultural safety and aims to promote intercultural dialogue between Indigenous and Western health care traditions. The study was part of a bigger initiative to foster intercultural dialogue [27], in which parties with different cultural backgrounds converge to work out solutions around a shared concern of poorer maternal health outcomes [28, 29] by respecting Indigenous skills and ways and recognising the needs of scientific evidence [30].

### Trial design

This pragmatic parallel-group cluster-randomised controlled trial tested non-inferiority of a co-designed intervention to support traditional midwifery in two intervention and two control municipalities between 2015 and 2017. Since ethnicity clusters and midwives serve a fixed community base, we used a cluster design. We describe the trial methods fully elsewhere [27]. The study included two levels of clustering: municipalities and communities within municipalities. Participating traditional midwives contributed to the design of the intervention by specifying what support they needed. We measured impact using an administered household survey, interviewing all women who reported pregnancy or childbirth in all involved municipalities during the study period. Trained bilingual Indigenous interviewers administered a baseline survey (February and March 2015) and a follow-up survey using the same procedure and questions (between April and May 2017). Reporting follows the CONSORT Statement [31] and its extensions to cluster randomised trials [32], non-inferiority trials [33] and abstracts [34].

### Ethics

The study conformed to specific ethical principles of research with Indigenous communities [35, 36] and obtained ethical approval from the Ethics Committee of the *Centro de Investigación de Enfermedades Tropicales* of the *Universidad Autónoma de Guerrero* (Reference 2013–014) and the Institutional Review Board of the Faculty of Medicine at McGill University (A06-B28-17B). The research team discussed the intervention with the leadership of all participating communities to get their approval to proceed in 2015. We obtained informed consent from Indigenous mothers during data collection and treated all responses from participants as confidential, with no identifying information recorded.

### Participants

Located in the south of Guerrero State in Mexico, the four municipalities are home to four Indigenous groups (*Nahua*, *Na savi*/Mixteco, *Me’phaa*/Tlapaneco and *Nancue ñomndaa*/Amuzgo). These Indigenous groups have different degrees of acculturation to the Western economy in Mexico, most still living in nuclear families in rural areas or remote small villages. They subsist on small-scale agriculture and migrant labour, typically receiving less than the average wage in the region (approximately $40 USD per month) [37]. The study included all Indigenous women in all 80 communities in the four municipalities who gave birth or who became pregnant during the study period (between 2015 and 2017) and their adult family members. There were no exclusions except people who left the municipalities, who were not followed.

The baseline survey identified 30 active traditional midwives (28 women and 2 men) whose communities recognised them based on the positive outcomes of their patients. They were mature adults with decades of practice, spoke almost exclusively Indigenous languages (*Me’phaa* and *Nancue ñomndaa*), used traditional techniques learned from a mentor traditional midwife, which included rituals and use of medicinal plants, and had strong connections with the community in which they lived.

### Intervention

The co-designed intervention supported traditional midwifery with four components [27]: *(i) Material support:* Authentic traditional midwives received a small stipend to increase the time available for their practice and patient care. *(ii) Apprentice support:* The project paid a scholarship for one apprentice for each midwife. Midwives each appointed their own apprentices and defined their training. Apprentices supported tasks some midwives could no longer perform due to age. *(iii) Sensitisation training for staff in the local government health centres*: CIET researchers led a workshop in each intervention municipality to present evidence about the role of traditional midwives and the importance of intercultural skills for Western medical practice. *(iv) Intercultural health brokers*: Community-appointed bilingual young community members received a two-month training (280 h) as *técnicos interculturales de salud*. Training covered primary health care, recovery and protection of Indigenous culture, and conservation of their territory. After the course, intercultural brokers went back to their communities and supported traditional midwives to increase their reach in the communities and to bridge their interaction with Western health personnel.

Control municipalities continued with usual health services as described in the protocol [27]. Most of the communities in these municipalities also had traditional midwives but they were not supported by the project.

### Primary outcomes

The questionnaire asked each household about household members, pregnancies and births. Women who had given birth in the last year, whether at home or in a health facility, responded to a questionnaire about their pregnancy and childbirth. Primary outcomes for comparison between intervention and non-intervention communities included: birth complications (breach position at birth, excessive bleeding, convulsions and retained placenta); perineal trauma (cut or tear) during childbirth; Caesarean section, and whether the wound became infected; and complications during birth affecting the health of the baby. We intended to ask about postpartum infection, but an error in administration of the questionnaire meant we did not collect this information.

### Secondary outcomes

Secondary outcomes reflected cultural safety. This implies non-disruption of traditional ways of Indigenous groups, which include use of traditional midwives and childbirth at home, in the presence of family. The secondary outcomes included: the number of antenatal check-ups by traditional midwives (none vs any); place of birth (home or facility); intended place for future births (home or facility); presence of family members at birth; and use of the traditional vertical position during labour.

Other secondary outcomes reflected interaction between traditional and Western care. We recorded the number of antenatal check-ups in Western care facilities (none vs any and up to four vs five or more). In Mexico, the recommendation is at least five antenatal care check-ups. We also recorded timing of the first antenatal care visit (whether during the first trimester or not) and asked if a traditional midwife had advised the pregnant woman to visit a health facility during their pregnancy for routine antenatal care. We categorised attendance at the birth as skilled birth attendance by a traditional midwife or a Western-trained health worker (community health worker, nurse or doctor), non-skilled (for example neighbours or family members), or none.

We asked women who gave birth in institutions about their perinatal management to indicate respect of cultural practices. The women reported if they chose the birth position, availability of translators if required, bathing in cold water, handling of the placenta, retention of amulets, and how respectful they considered their management.

We asked specific questions to explore secondary outcomes of factors that could negatively affect maternal health like heavy work late in pregnancy and food intake during pregnancy. Women also reported the costs of transportation to reach Western health care facilities and if they paid for childbirth, either at home or elsewhere.

### Sample size

Interpretation of non-inferiority trials rests on the upper limit of the effect measure confidence interval [33]. Study power calculation is thus to detect a minimum margin of non-inferiority. We used the ClusterPower package in R [38]: across 80 communities in four municipalities and a between cluster variability of 0.05, a baseline rate of 30% for the occurrence of any of the primary outcomes (serious childbirth complications, perinatal deaths and neonatal complications), 420 births in each arm would permit a power of 86% to exclude a difference in favour of the control group (margin of non-inferiority) of more than 15% as the upper limit of a 95% two-sided confidence interval. In the absence of previous studies and estimations of hierarchical variability between municipalities and communities, our calculation recognises the study size needed to establish non-inferiority in the presence of small adverse effects of the intervention and high variability of such effects across clusters. Hierarchical models with more than two levels of clustering would shrink parameters towards the parameters of upper levels [39]. Without previous studies, any assumption on the direction of shrinkage would be speculative.

In practice, the trial included all the available pregnant women in the participating communities for the duration permitted by the existing funding. In the Discussion, we comment on the limitations in interpretation related to sample size.

### Randomisation and masking

An epidemiologist not involved in the fieldwork (NA) generated the allocation sequence and assigned the four municipalities into two parallel groups. The CIET team led by SPS oversaw enrolment of clusters. There was no possibility to conceal intervention status from communities once the intervention began, as supporting traditional midwifery was clearly different from pre-intervention status with unsupported traditional midwifery.

### Statistical methods

The primary analysis reported outcomes as absolute event rates among intervention and control groups, risk difference (difference of exposed and control rates) with two-sided 95% confidence intervals (95%CI) [40]. To establish compatibility of our data with the hypothesis of non-inferior performance of the intervention arm [41], we calculated the significance level at which the confidence interval would exclude parity. We also reported the equivalent odds ratios (OR), a more common measure of effect for binary data [40], to support interpretation. Risk difference is useful for discussion of public health impacts of the intervention and to indicate how the risk of a group would change with the intervention. The interpretation of the OR would be more useful to inform mothers on how supporting traditional midwives could change their chance of having a bad health outcome. To calculate the intra-cluster correlation coefficient (ICC), the open-source software CIETmap 2.2 [42] divided the between-cluster variance by the variance within and between clusters. The primary analysis followed intention-to-treat principles (everyone included in each cluster, per allocation). A cluster-level analysis used the Welch modification of the t-test [40] to compare the mean of the cluster event rates in the intervention and control arms to incorporate the variability across communities. The calculation included the baseline imbalances of the outcomes summarised at the cluster level [43]. The analysis compared cluster-level effects at the community level to account for the maximum variability of both intervention and effect measures.

### Secondary analysis

We established cluster-level differences of primary and secondary outcomes with a multilevel analysis using generalised linear mixed modelling (GLMM) with community as a random effect [44]. The regression models to calculate the measures of effect included differences at baseline. For each model, we reported OR with 95%CI.

### Sensitivity analysis

The intervention intended to implement all components in all intervention sites. In practice, community security in the face of narco-traffic activities and other factors led to a range of implementation fidelity. To establish the level of fidelity in each community, participating traditional midwives each scored the four intervention components in their communities before the analysis of the final survey (July 2018). The lead author analysed the results of the fidelity scores after the final survey but blind to the results of the survey. We used a classification tree available in *rpart* in R to identify baseline characteristics associated with differences in implementation fidelity [45].

A methodological concern in non-inferiority trials using an intention to treat analysis is the potential dilution of the effect measure simply because a participant might have not received the intervention [46]. If the tested treatment was inferior, the observed negative effect in an intention to treat analysis could be smaller than if all patients had adhered to treatment, conversely the study would be more conservative to establish superior effects [33]. We tested consistency across levels of fidelity with three additional analyses [33, 47]. First, we compared outcomes in fully protocol-adherent communities with those in control communities, using GLMM with community as a random effect, adjusted by baseline characteristics. Second, we considered four categories of implementation: (1) communities with good performance in all four intervention components, (2) those with good performance in three components, (3) those with good performance in less than three components and (4) control communities. Finally, we used an instrumental variable analysis to establish the effect among compliers as the ratio of the ITT analysis estimate to the proportion of compliers [48, 49].

### Missing data

We report the proportion of missing data for each outcome. We characterised subjects with missing data as far as possible and analysed the effect of missing data using the multiple imputation method in the Amelia II programme in R. We produced ten imputed databases and compared the results with the complete case analysis.

## Results

**Fig. 1:**
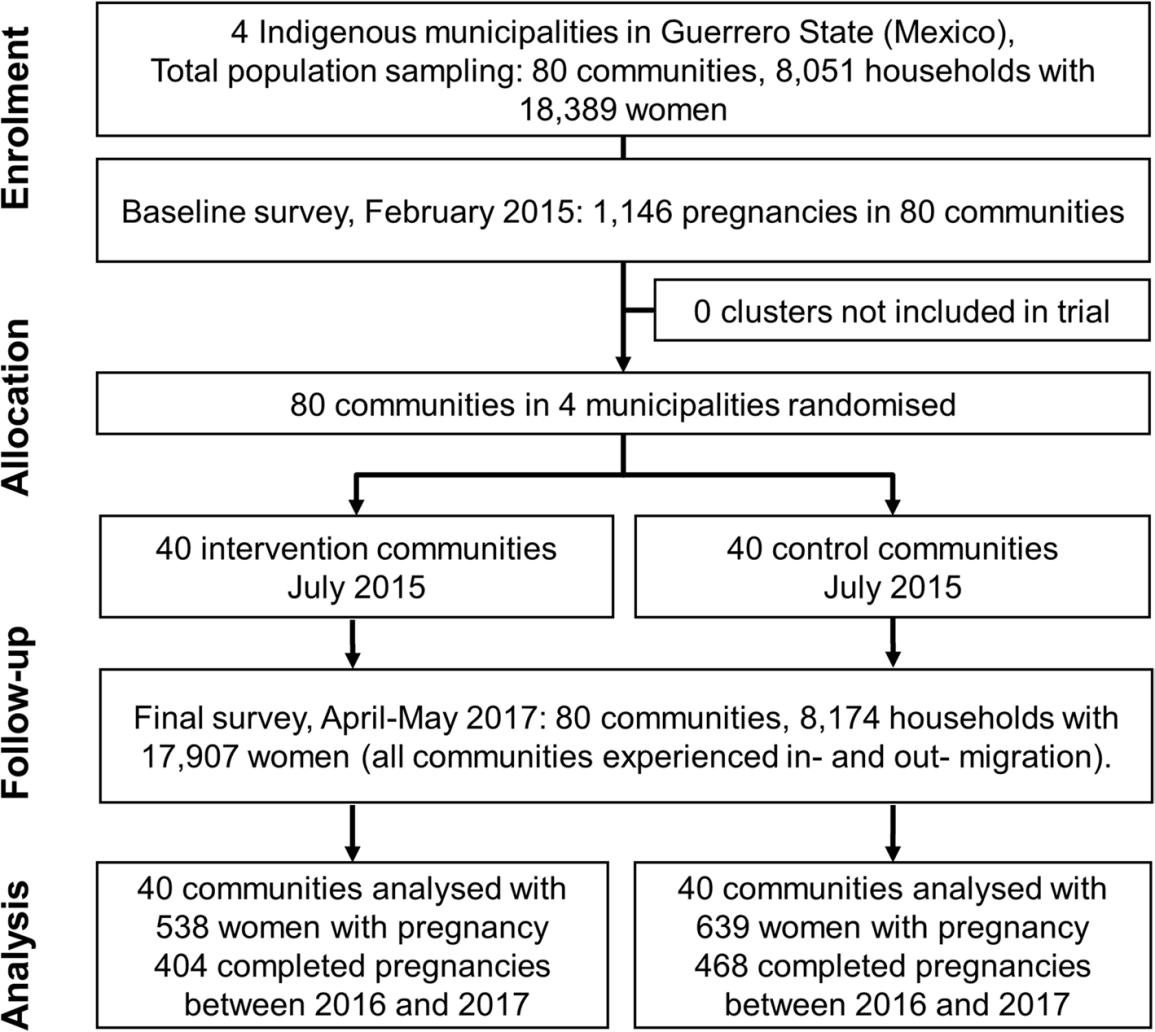
Flow diagram of progress of clusters and individuals through phases of the randomised trial

Figure 1 shows the participant flow of 18,389 women, 6168 of them aged 14-49 years in 8051 households in 80 community clusters through the trial. The intervention began in July 2015, with the final survey between April and May 2017, at which time there were 17,907 women (6188 aged 14-49 years) in 8174 households. All communities experienced in-migration and out-migration. We added new arrivals to the study but did not follow those leaving the clusters, many of whom migrated to the cities. The final survey included 1177 women who were pregnant during the last year in the intervention and control municipalities; 872 of them reported having completed their pregnancy and 305 were still pregnant (average gestational age 5.9 months, standard deviation 2 months). Nine households reported two pregnant women in the study period.

Table 1 shows 2015 baseline characteristics of included women in the intervention and control arms. Fewer women in the intervention communities used Spanish (they spoke only Indigenous languages). Women in intervention communities were also less likely to have attended school and more likely to be single, to have had their last birth at home, without skilled attendance, and to have made payments related to the birth.

**Table 1:** Baseline characteristics of intervention and control arms in 2015

Variable	Absolut event rates (n)	
	Intervention (cluster *n* = 40)	Control (cluster *n* = 40)
Interviewed households (HH)	0.47 (3756/8051)	0.53 (4295/8051)
HH without tap water	0.14 (518/3704)	0.22 (928/4246)
Among 1146 pregnancies		
Woman’s age^a^	25.7 (0.4, *n* = 528)	25.9 (0.4, *n* = 599)
Woman speaks Spanish (*p* = 0.00)^b^	**0.69 (355/512)**	**0.86 (501/580)**
Woman’s education above primary (*p* = 0.02)^b^	**0.45 (237/529)**	**0.56 (329/593)**
Women receives government aid	0.64 (337/525)	0.66 (388/591)
Woman has health insurance	0.93 (491/527)	0.92 (548/593)
Woman without a partner (*p* = 0.05)^b^	**0.07 (35/529)**	**0.10 (59/596)**
Among 841 completed pregnancies		
Perinatal deaths	0.03 (11/386)	0.03 (14/448)
Neonatal complications	0.17 (61/362)	0.18 (76/427)
Childbirth serious complications	0.20 (74/366)	0.17 (72/437)
Any primary outcome^c^	0.33 (128/389)	0.31 (140/452)
Woman is main decision maker (alone or with partner)	0.67 (248/372)	0.74 (326/439)
Traditional midwife saw the woman at least once	0.75 (282/378)	0.71 (313/441)
Woman went to WHC for antenatal care	0.97 (357/369)	0.97 (422/434)
Gestational age of first recourse to WHC^a^	3.2 (0.1, *n* = 380)	2.8 (0.1, *n* = 441)
Childbirth at home (*p* = 0.00)^b^	**0.46 (171/369)**	**0.26 (115/438)**
Unattended childbirth^d^ (*p* = 0.05)^b^	**0.08 (28/375)**	**0.04 (17/439)**
Childbirth with traditional midwife (*p* = 0.02)^b^	**0.37 (137/375)**	**0.23 (99/439)**
Woman paid for childbirth (*p* = 0.00)^b^	**0.43 (141/332)**	**0.29 (121/412)**
Company of family during childbirth (*p* = 0.00)^b^	**0.75 (275/368)**	**0.43 (189/437)**
Traditional childbirth (*p* = 0.01)^b^	**0.26 (98/376)**	**0.13 (58/441)**
Woman did not suffer violence during pregnancy	0.97 (361/372)	0.97 (427/441)
Infected wound after childbirth (*p* = 0.03)^b^	**0.06 (21/343)**	**0.03 (11/422)**

Missing data ranged between 0 and 11.5%; the highest proportion of missing data was for Woman paid for childbirth (11.5% or 97/841 completed pregnancies) and Infected wound after childbirth (9% or 76/841)

*HH* Household, *WHC* Western health care facility

^a^Average (SD, n)

^b^cluster-level t-test

^c^childbirth or neonatal complication or perinatal death

^d^Medical doctor, nurse or traditional midwife vs casual or unattended childbirth

The baseline survey (Table 1) showed very similar rates of perinatal deaths and neonatal complications between intervention and control communities. It suggested more childbirth complications in communities that became the intervention arm, although this was not statistically significant at the 5% level.

Table 2 shows the demographic characteristics of women in intervention and control communities in 2017. Women in the intervention communities were significantly less likely to have received formal education (RD -0.22 95%CI − 0.31 to − 0.13) and to speak Spanish (RD -0.14 95%CI − 0.23 to − 0.05). Missing data of demographic characteristics varied between 1.6 and 3%. We could not detect any identifiable pattern for missing data. The comparison of complete case analysis with imputed datasets did not significantly differ with proportions reported in Table 2.

**Table 2:** Demographic characteristics of intervention and control arms in 2017

Variable	Absolut event rate (n)	
	Intervention (cluster *n* = 40)	Control (cluster *n* = 40)
Interviewed households (HH)	0.47 (3836/8174)	0.53 (4338/8174)
Total population	0.47 (16,321/35091)	0.54 (18,770/35091)
People in each HH*	4.3 (2.0, *n* = 3836)	4.3 (2.0, *n* = 4338)
HH without tap water	0.88 (3351/3807)	0.81 (3518/4321)
Women parity*	2.0 (1.7, *n* = 530)	1.9 (1.7, *n* = 628)
Women age*	25.5 (6.6, *n* = 530)	25.6 (6.6, *n* = 627)
Women education above primary (*p* = 0.00)^a^	**0.47 (246/529)**	**0.60 (372/622)**
Woman speaks Spanish (*p* = 0.00)^a^	**0.68 (353/521)**	**0.90 (556/621)**
Woman without a partner	0.07 (37/527)	0.07 (45/623)
Women receives government aid	0.58 (304/524)	0.53 (329/620)
Woman has health insurance	0.94 (497/529)	0.94 (586/624)

*Average (standard deviation SD, n)

^a^cluster-level t-test

## Outcomes and estimation of the impact

### Primary outcomes

We analysed event rates of perinatal deaths, mother’s report of neonatal complications and serious birth complications between 2016 and 2017 on an intention-to-treat basis. Data was compatible at the 95% level with a lower risk of having one or more of the three primary outcomes (perinatal deaths or childbirth or neonatal complications) in the intervention communities (RD -0.06 95%CI − 0.09 to − 0.02) (Table 3).

**Table 3:** Effect measures for the primary outcomes (intention to treat analysis)

Variable	Absolut event rate (n) (cluster *n* = 40)		RD 95%CI^a^	OR 95%CI^b^	ICC
	Intervention	Control			
Any primary outcomes^c^	0.12 (50/404)	0.18 (85/468)	**− 0.06** **− 0.09 to − 0.02**	**0.63** **0.42 to 0.95**	0.03
Perinatal deaths 2016 and 2017	0.02 (10/404)	0.03 (16/468)	− 0.01 − 0.04 to 0.02	0.73 0.31 to 1.75	0.04
Neonatal complications^d^	0.06 (23/389)	0.07 (39/456)	− 0.04 − 0.08 to 0.01	0.59 0.30 to 1.18	0.05
Childbirth serious problems	0.05 (19/393)	0.08 (35/456)	**− 0.05** **− 0.08 to − 0.02**	**0.35** **0.14 to 0.92**	0.00
Caesarean section	0.13 (50/381)	0.15 (66/441)	−0.03 − 0.10 to 0.04	0.78 0.47 to 1.31	0.11
Perineal trauma or wound infection	0.21 (81/389)	0.21 (94/455)	0.01 −0.07 to 0.08	1.06 0.70 to 1.61	0.07
Perineal trauma or wound infection in home childbirths	0.11 (17/156)	0.24 (27/116)	−0.12 − 0.27 to 0.01	0.40 0.15 to 1.05	0.18

*RD* risk difference, *OR* odds ratio, *ICC* Intra-cluster correlation coefficient

^a^Baseline-adjusted cluster-level analysis using t-test as presented by Campbell, 2014

^b^OR and confidence intervals calculated with a GLMM using lme4 package in R

^c^childbirth or neonatal complication or perinatal death

^d^854 women with completed pregnancies reported on childbirth or neonatal complications, and 18 women with completed pregnancies reported only on perinatal deaths

Participants reported a total of 26 perinatal deaths in the households. There was a suggestion of reduced risk of perinatal deaths and neonatal complications in intervention communities compared with control communities, but the differences were not significant at the 5% level (Table 3). The risk of serious childbirth complications was significantly lower in intervention communities (RD -0.05 95%CI − 0.08 to − 0.02). The analysis excluded parity in favour of the intervention with 95% confidence for childbirth complications (RD 95%CI − 0.08 to − 0.02), 90% confidence for neonatal complications (RD 90%CI − 0.07 to 0.00) and 70% confidence for perinatal mortality (RD 70%CI − 0.02 to 0.00).

Adjusted for baseline differences of the outcomes, a GLMM with intervention as a fixed effect and community as a random effect showed very similar measures of effect as the unadjusted analysis (shown in Table 3).

Overall, women in intervention communities had almost identical rates of postpartum perineal trauma or wound infection (Table 3). However, among home childbirths, women in intervention communities had a lower risk of perineal trauma or wound infection compared with women in control communities, although this was not significant at 5% level.

Missing data of primary outcomes varied from 0.6% (5/854 for childbirth serious complications) to 3.7% (32/854 for Caesarean section). We could not identify particular patterns for missing data as the numbers of missing data subjects were sparse. Comparison of imputed datasets and complete case analysis did not identify significant divergences, but showed increased uncertainty around the difference for the number of Caesarean sections.

### Secondary outcomes

Analysis of secondary outcomes confirmed higher rates of traditional birth (at home, in company of family, with traditional midwife and mainly in vertical position) in intervention communities (RD 0.10 95%CI 0.02 to 0.18) (Table 4). More births were attended by traditional midwives in intervention than in control communities (RD 0.14 95% 0.03 to 0.25) (Table 4). In both intervention and control communities, traditional midwives saw more than 70% of all women during pregnancy, and almost 70% of those who gave birth in Western healthcare facilities (Table 4).

**Table 4:** Secondary outcomes (intention to treat analysis)

Secondary outcomes	Absolute event rate (n) (cluster *n* = 40)		RD 95%CIa^a^	OR 95%CI^b^
	Intervention	Control		
*Traditional childbirth*				
Total traditional childbirths ^c d^	0.19 (75/394)	0.11 (48/457)	**0.10** **0.02 to 0.18**	**2.95** **1.27 to 6.84**
Childbirth with traditional midwife ^c^	0.31 (123/392)	0.20 (90/456)	**0.14** **0.03 to 0.25**	**2.49** **2.20 to 5.17**
Childbirth at home	0.40 (158/394)	0.25 (114/454)	0.04 − 0.11 to 0.19	1.13 0.52 to 2.45
Vertical childbirth^c^	0.29 (111/388)	0.18 (81/454)	**0.12** **0.03 to 0.22**	**2.38** **1.26 to 4.47**
Company of family during childbirth ^c^	0.63 (244/390)	0.46 (205/450)	**0.17** **0.06 to 0.28**	**2.21** **1.41 to 3.48**
Intention of future childbirth at WHC ^c^	0.64 (244/379)	0.80 (362/453)	**−0.17** **− 0.26 to − 0.07**	**0.37** **0.21 to 0.66**
Traditional midwife saw the woman at least once	0.78 (406/523)	0.73 (458/624)	0.06 −0.02 to 0.14	1.47 0.93 to 2.33
*Access to health care services*				
Woman went to WHC for antenatal care	0.95 (497/523)	0.96 (590/618)	0.00 −0.04 to 0.03	1.04 0.49 to 2.22
At least 5 check-ups with WHC	0.71 (264/376)	0.74 (323/437)	−0.03 − 0.12 to 0.05	0.88 0.57 to 1.36
Gestational age of first recourse to WHC*	3.0 (0.3, *n* = 392)	3.0 (0.1, *n* = 459)		
Traditional midwife advised visiting WHC	0.79 (246/310)	0.82 (292/356)	−0.06 −0.19 to 0.07	0.87 0.55 to 1.37
Childbirth with Western provider ^e^	0.61 (238/392)	0.75 (340/456)	− 0.01 − 0.16 to 0.11	0.88 0.39 to 1.99
Antenatal care with traditional midwife and childbirth with Western provider	0.68 (161/237)	0.70 (237/340)	− 0.02 − 0.12 to 0.08	1.09 0.66 to 1.82
Unattended childbirths	0.08 (31/392)	0.06 (26/456)	0.02 −0.02 to 0.06	1.58 0.74 to 3.35
Paid for childbirth	0.38 (138/367)	0.24 (106/443)	0.12 − 0.02 to 0.25	1.83 0.95 to 3.52
Paid for childbirth with Western provider ^c^	0.28 (62/225)	0.13 (44/331)	**0.14** **0.04 to 0.24**	**2.67** **1.26 to 5.66**
Average cost of childbirth (USD)*	68.6 (28.7, *n* = 138)	84.3 (14.7, *n* = 106)		
*Childbirth in Western facility*				
The woman chose childbirth position	0.60 (139/231)	0.61 (205/336)	−0.08 − 0.24 to 0.08	0.97 0.57 to 1.66
Translator during childbirth (if needed)	0.42 (63/151)	0.32 (83/262)	0.04 −0.09 to 0.18	1.59 0.94 to 2.69
Woman had to bathe with cold water ^c^	0.60 (138/231)	0.45 (154/341)	**0.10** **0.02 to 0.19**	**1.72** **0.99 to 3.00**
The woman received the placenta^ c^	0.56 (130/232)	0.21 (69/335)	**0.34** **0.21 to 0.48**	**6.15** **3.24 to 11.7**
Retention of amulets	0.30 (68/229)	0.27 (89/335)	0.03 −0.09 to 0.15	1.18 0.67 to 2.06
The woman felt she was treated with respect	0.89 (210/237)	0.92 (312/338)	−0.04 − 0.12 to 0.03	0.67 0.31 to 1.42
*Behaviours*				
Woman eats same or more than usual during pregnancy	0.22 (85/394)	0.28 (125/453)	−0.05 − 0.11 to 0.02	0.74 0.50 to 1.10
Woman works less than usual during pregnancy	0.37 (144/394)	0.39 (177/457)	−0.01 − 0.10 to 0.09	0.90 0.62 to 1.30

*WHC* Western health care facility, *RD* risk difference, *OR* odds ratio

^a^Baseline-adjusted cluster-level analysis using t-test as presented by Campbell, 2014

^b^OR and confidence intervals calculated with a GLMM using lme4 package in R

^c^significant differences at the 5% level

^d^at home, with traditional midwife and family and in preferred vertical position

^e^doctor, nurse or health promotor

*Average (standard deviation SD, n)

Women in intervention communities were also significantly less likely to say they intended to have future hospital-based childbirth (RD -0.17 95%CI − 0.26 to − 0.07) (Table 4). This preference was associated with the place of the last childbirth. Within intervention communities, a woman who gave birth with a traditional midwife was less likely to desire a future institutional childbirth (31.4% or 38/121) compared with a woman who had institutional childbirth (80.5% or 206/256, OR 0.13 95%CI 0.06 to 0.21). Among those who gave birth in Western health facilities, women in intervention communities were significantly more likely to receive the placenta after childbirth, as required by custom, than women in the control arm (RD 0.34 95% 0.21-0.48) (Table 4). There were no other positive differences in the experience in health care facilities of women in intervention communities, and they were more likely to report being forced to bath with cold water after childbirth (RD 0.10 95% 0.02 to 0.19) (Table 4). For childbirths with a Western provider, more intervention women had to pay than did control women (RD 0.14 95%CI 0.04 to 0.24).

### Sensitivity analysis

The traditional midwives reported that 13/40 communities performed well across the four intervention components, 9/40 performed well in three components, and 18/40 performed well in less than three components (Supplementary material 1). The main concerns of traditional midwives regarding implementation were the commitment of some apprentices and the lack of continuity of five intercultural brokers. Communities with a smaller number of births at home and remote communities with less Indigenous language speakers and communities with a Western health facility had lower intervention fidelity. Per-protocol analysis compared the communities with the highest level of intervention fidelity (29.4% or 119/404 childbirths) with the control communities. Women in protocol-adherent communities showed higher rates of traditional births than women in control communities (RD 0.23 95%CI 0.09 to 0.38) (Table 5). These communities also had fewer unattended childbirths than did control communities (difference not significant at the 5% level, Table 5). Most of the unattended childbirths observed in the intervention arm happened in communities with less than three intervention components satisfactorily implemented (5.1% (10/197) compared with 10.8 (21/195), RD -0.06 95%CI − 0.11 to − 0.01).

**Table 5:** Sensitivity analyses incorporating levels of fidelity to the intervention

	Absolut event rate (n)		RD 95%CIa ^a^	OR 95%CI ^b^
	Intervention	Control		
*Protocol-adherent communities* vs *control communities*	(cluster *n* = 13)	(cluster *n* = 40)		
Total traditional childbirths ^c d^	0.34 (40/119)	0.11 (48/457)	**0.23** **0.09 to 0.38**	**8.67** **2.70 to 27.8**
Unattended childbirths	0.03 (4/117)	0.06 (26/456)	−0.02 −0.08 to 0.03	0.69 0.18 to 2.72
Perinatal mortality	0.03 (3/122)	0.03 (16/468)	−0.02 −0.06 to 0.03	0.66 0.25 to 1.77
Neonatal complications	0.06 (7/118)	0.09 (39/456)	−0.03 −0.10 to 0.04	0.61 0.25 to 1.71
Childbirth complications ^c^	0.03 (3/119)	0.08 (35/456)	**−0.05** **−0.08 to −0.02**	**0.35** **0.14 to 0.92**
*As treated*	(cluster *n* = 22)	(cluster *n* = 40)		
Childbirth complications Fear or good performance vs control ^c^	0.03 (6/199)	0.08 (35/456)	**−0.05** **−0.08 to −0.01**	**0.37** **0.15 to 0.90**
		(cluster *n* = 58)		
Childbirth complications Fear or good vs control and poor performance ^c^	0.03 (6/199)	0.07 (48/650)	**−0.04** **−0.08 to −0.01**	**0.39** **0.16 to 0.93**
*Instrumental variable*	(cluster *n* = 40)	(cluster *n* = 40)		
Perinatal mortality	0.03 (3/122)	0.03 (16/468)	−0.03 −0.12 to 0.06	
Neonatal complications	0.06 (7/118)	0.09 (39/456)	−0.11 − 0.25 to 0.04	
Childbirth complications ^c^	0.03 (3/119)	0.08 (35/456)	**−0.09** **−0.18 to 0.00**	

*RD* risk difference, *OR* odds ratio

^a^Baseline-adjusted cluster-level analysis using t-test as presented by Campbell, 2014

^b^OR and confidence intervals calculated with a GLMM using lme4 package in R

^c^Significant differences at the 5% level

^d^at home, with traditional midwife and family and in preferred vertical position

The measures of effect for primary outcomes confirmed the results from the intention to treat analysis with wider confidence intervals, due to a reduced number of participants involved in the calculation. When compared with control communities (Table 5), protocol-adherent communities had non significantly lower perinatal mortality, neonatal complications and significantly lower childbirth complications (RD -0.05 95%CI − 0.08 to − 0.02).

Supplementary material 2 shows a comparison of groups as treated using GLMM. Serious complications were significantly lower in the communities with fair or good performance compared with control communities (RD -0.05 95%CI − 0.08 to − 0.01) or compared with control and poor performance communities together (RD -0.04 95%CI − 0.08 to − 0.01) (Table 5).

The instrumental variable analysis confirmed the average protective effect among compliers although with increased confidence intervals for perinatal deaths and neonatal complications (Table 5). For serious childbirth complications, this analysis also confirmed exclusion of inferior performance of the intervention among compliers (RD − 0.09 95%CI − 0.18 to 0.00).

## Discussion

The Safe Birth in Cultural Safety trial in Guerrero found lower perinatal deaths, neonatal complications and serious childbirth complications after 21 months of supporting traditional midwives on their own terms. Two-sided 95% confidence intervals excluded parity for childbirth complications, and indicated non-inferiority limits of 1 and 2% for neonatal complications and perinatal deaths, respectively. Consistent results for ITT and non-ITT analyses confirmed lower rates of primary outcomes in those communities with higher fidelity intervention.

Non-inferiority trials are intended to show the intervention is not worse than the existing option on the premise that it has other advantages [33]. Supporting traditional midwives on their own terms improved cultural safety by increasing traditional childbirths and, in communities where there was higher intervention fidelity, lowering rates of unattended births. The intervention improved handling of the placenta in institutional births, to accommodate traditional norms. Other aspects of cultural safety in Western facilities failed to improve.

For almost a century [50], engagement of traditional midwives has focused on their being re-trained and used as auxiliary health workers to extend the provision of Western health services [3, 51–53]. Apart from our pilot trial [27], we could not identify any published trial of supporting traditional midwifery on its own terms.

Several studies report positive effects on peri-neonatal morbidity and mortality from working with traditional midwives [10, 11, 13–15, 19, 54], some exploring cost-effective results [21, 22]. Traditional midwives in Guerrero described complex knowledge of risk factors and preventive practices, albeit framed in the terms of their traditional culture [55]. In a similar context in Guatemala, Austad [56] reported improvements in management of complications associated with support of obstetric care navigators, a role that intercultural brokers in Guerrero offered in coordination with the traditional midwives and their apprentices [57].

For many Indigenous communities, place of birth and involvement with childbirth rituals are connected to identity, culture and territories, and even some roles in governance [58, 59]. In our study, women who gave birth at home with a traditional midwife were less likely to intend to have an institutional childbirth in the future. Preference for home births in this region is closely linked to cultural values, and mistreatment or disrespect shown to Indigenous women in health institutions reinforces this preference [26, 60, 61]. Beyond Indigenous communities, mistreatment [62], disrespect [63], and violence against women [64] during childbirth have gained increasing attention [65, 66]. Despite the controversy surrounding the safety of home births [67–69], our study supports the argument that safe birth in places like Guerrero “needs a fully integrated comprehensive maternity care network that is supportive and responsive” [70]. In a context like ours, where iIdigenous women mostly give birth at home [26], informed and principled interaction of the official health system with traditional midwives can pave the way for respectful and women-centred care.

Notwithstanding the well-documented benefits of modern obstetric care for the medical safety of mothers and children, there are also unintended side effects and iatrogenic illnesses [71, 72]. There are some procedures that women consider injurious but providers do not [63, 65]. Understanding what Indigenous women consider harmful practices requires interaction and mutual learning [30]. Rituals associated with handling of the placenta, for example, have profound implications for cultural identity in these communities and set the path for a healthy life of the child. Baths with cold water in the postpartum period, on the other hand, are regarded as violence, a source of *coldness of the womb,* and a cause of poor maternal health [55]. Promotion of cultural safety in Western institutions requires additional efforts and is an ongoing challenge for medical education.

### Limitations and strengths

Sample size is a common limitation of research with small and remote Indigenous communities even including, as we did, all the women in the community. Accumulating numbers of events by increasing the duration of the study depends on availability of funding. We reported on 872 completed pregnancies and some additional months of follow up would have allowed us to include in the analysis the outcomes of several hundred women who were still pregnant at the moment of the final survey. The cluster design avoided contamination that would occur if intervention traditional midwives attended women in control communities [73]. The clustered design reduced the power of the study, making it harder to demonstrate non-inferiority. The baseline survey in 2015 revealed intervention women had important differences from control women in their language, schooling and support during childbirth. The direction of the differences would have us expect worse maternal health outcomes in intervention communities, which could lead to underestimation of a positive effect of the intervention.

The difficult field conditions affected measurement of gender violence, a key ripple effect in the pilot study. Interviewers had to administer these questions, for security reasons, under conditions where the respondent could be seen and possibly overheard. We observed similar difficulties and attendant limits to interpretation earlier in Guerrero [74] and in Pakistan [75].

Knowledge of intervention status could have affected some secondary outcomes (for example, intention of future home-based childbirths). The main outcome indicators (non-inferiority for perinatal mortality, neonatal and serious childbirth complications) and other secondary outcomes would be less susceptible to this bias.

The study benefitted from decades of institutional commitment and experience of CIET researchers that cannot be assumed in other contexts. This community engagement generated the co-designed intervention. During the trial, the researchers also generated institutional support in government facilities, and established a favourable environment to discuss results with local authorities.

## Conclusions

Supporting traditional midwives on their own terms can increase cultural safety without worsening birth outcomes. The small size of Indigenous populations and restricted funding for the intervention limits interpretation of this potentially important finding. Further research needs to explore the added benefit of increased collaboration with Western stakeholders. Traditional midwifery could contribute to safer birth among Indigenous communities if, instead of attempting to replace traditional practices, health authorities promoted intercultural dialogue.

## Supplementary Information

**Additional file 1.** Evaluation of the implementation of the intervention and baseline characteristics. Evaluation of each of the components of the intervention according to the participating traditional midwives and baseline characteristics of each community.

**Additional file 2.** Comparison of groups as treated using GLMMs. The table shows the comparison of groups as treat using OR and 95% confidence intervals.

## Data Availability

The datasets used and/or analysed during the current study are available from the corresponding author on reasonable request. According to the agreements with participating communities, to ensure the protection of participants and governance of data, before the information can be shared, the requester will need to present a plan for data analysis. Also, the requester will need to complete the procedure for ethical approval of the secondary analysis in accordance with the procedures defined by the Ethics Board of the *Universidad Autónoma de Guerrero*.

## Abbreviations

CI: Confidence interval
CIET: Centro de Investigación de Enfermedades Tropicales
CINAHL: Cumulative Index to Nursing and Allied Health Literature
CONSORT: Consolidated Standards of Reporting Trials
GLMM: Generalised linear mixed model
ICC: Intra-cluster correlation coefficient
ITT: Intention to treat
LILACS: Latin American and Caribbean Health Sciences Literature
OR: Odds ratio
RCT: Randomised controlled trial
RD: Risk difference
