# Supplementary material for: Safe birth in cultural safety in southern Mexico: a pragmatic non-inferiority cluster-randomised controlled trial
Source: BMC Pregnancy Childbirth. 2022 Jan 17;22:43. doi: 10.1186/s12884-021-04344-w (PMC8762841; doi:10.1186/s12884-021-04344-w)
Supplement: Supplementary file 1 — Additional file 1. Evaluation of the implementation of the intervention and baseline characteristics. Evaluation of each of the components of the intervention according to the participating traditional midwives and baseline characteristics of each community. [file 12884_2021_4344_MOESM1_ESM.docx]

**Supplementary material 1**

**Evaluation of the implementation of the intervention and baseline characteristics of the communities**

|  | Evaluation of the intervention | | | | | Proportion of households with the characteristic according to the baseline survey | | | | | | | | |
| --- | --- | --- | --- | --- | --- | --- | --- | --- | --- | --- | --- | --- | --- | --- |
| Community | As  treated | Per Protocol  status | Traditional  Midwife | Intercultural  Broker | Apprentice | Spanish speakers | Domestic violence | Women decide | Skilled attend. | Home deliveries | Intention of future hospital deliveries | Health facility in the community | Tap water | Remote |
| Cluster#1 | | | | | | | | | | | | | | |
| 7101 | A | Int. | 1 | Fair | Good | Low | High | High | High | Low | High | Yes | High | No |
| 7102 | C | No Int. | visiting TM | Bad* | Bad | High | Low | High | High | Low | High | Yes | High | No |
| 7103 | C | No Int. | visiting TM | Bad* | Bad | High | Low | Low | High | Low | High | Yes | High | No |
| 7104 | C | No Int. | 1^a^ | Bad* | Good | Low | High | Low | Low | Low | High | Yes | High | No |
| 7105 | C | No Int. | Same 7104 | Bad* | Good | Low | Low | Low | Low | Low | High | Yes | High | No |
| 7106 | B | No Int. | 1 | Good | Bad | High | High | High | Low | Low | High | Yes | High | No |
| 7107 | B | No Int. | Same 7106 | Good | Bad | Low | Low | Low | Low | Low | High | Yes | High | No |
| 7108 | C | No Int. | visiting TM | No | No | High | Low | High | High | Low | High | Yes | High | Yes |
| 7109 | B | No Int. | 1 | Bad | Good | Low | High | Low | Low | High | Low | Yes | High | No |
| 7110 | B | No Int. | 1 | Bad | Good | Low | High | High | Low | High | Low | No | High | No |
| 7111 | C | No Int. | 1 | Bad* | Good | Low | Low | NA | High | Low | High | No | High | No |
| 7112 | B | No Int. | Same 7101 | Fair | Fair | Low | Low | NA | High | High | Low | No | High | No |
| 7113 | C | No Int. | 1^a^ | Bad* | Good | High | Low | Low | High | Low | High | Yes | High | No |
| 7114 | A | Int. | 1 | Good | Bad | Low | Low | High | Low | High | High | No | Low | Yes |
| 7115 | A | Int. | 1 | Good | Bad | Low | Low | Low | High | High | Low | No | High | Yes |
| 7116 | C | No Int. | visiting TM | No | No | Low | Low | Low | Low | High | Low | No | Low | Yes |
| 7117 | C | No Int. | 1 | Bad* | Good | Low | High | Low | Low | High | Low | Yes | High | Yes |
| 7118 | C | No Int. | 1 | Bad* | Good | Low | Low | High | High | Low | High | No | High | No |
| 7119 | C | No Int. | 1 | Bad* | Good | Low | High | High | High | High | Low | No | High | No |
| 7120 | C | No Int. | 1 | Bad* | No | Low | High | Low | High | Low | High | No | High | No |
| Cluster#2 | | | | | | | | | | | | | | |
| 7601 | A | No Int. | 1 | Fair | Good | Low | High | High | High | Low | High | Yes | High | No |
| 7602 | A | No Int. | 1 | Good | Good | Low | Low | High | Low | High | Low | Yes | High | No |
| 7603 | C | No Int. | 1 | Good | Good | Low | Low | Low | Low | Low | Low | Yes | Low | No |
| 7604 | A | Int. | visiting TM | Bad | No | High | High | High | High | Low | Low | Yes | Low | No |
| 7605 | A | Int. | 1 | Good | Good | High | Low | Low | Low | High | Low | Yes | High | No |
| 7606 | A | Int. | 1+2 | Good | Fair | Low | Low | High | High | High | Low | Yes | High | No |
| 7607 | A | Int. | 1+4 | Excellent | Fair | High | Low | High | High | High | High | Yes | High | No |
| 7608 | A | Int. | 1 | Good | Fair | High | Low | Low | High | High | Low | Yes | Low | No |
| 7609 | B | No Int. | 1 | Good | Fair | Low | Low | High | Low | High | Low | Yes | Low | No |
| 7610 | C | No Int. | 1 | Bad | Good | High | Low | Low | High | High | Low | Yes | Low | No |
| 7611 | A | Int. | visiting TM | No | No | Low | Low | High | Low | High | Low | Yes | High | Yes |
| 7612 | B | No Int. | 1 | Fair | Good | Low | Low | Low | Low | High | Low | No | Low | No |
| 7613 | A | Int. | 1 | Bad | Fair | Low | Low | Low | Low | High | High | No | Low | No |
| 7614 | C | No Int. | 1^a^ | Good | Fair | High | Low | High | Low | Low | High | No | High | No |
| 7615 | B | No Int. | 1 | No | Fair | High | Low | Low | High | High | Low | No | High | No |
| 7616 | C | No Int. | 1 | Fair | Bad | Low | Low | Low | Low | High | Low | No | Low | Yes |
| 7617 | C | No Int. | visiting TM | No | No | High | High | High | High | Low | High | Yes | High | No |
| 7618 | C | No Int. | visiting TM | No | No | Low | Low | High | Low | Low | Low | Yes | Low | No |
| 7619 | B | No Int. | 1^a^ | No | Good | High | Low | High | High | High | High | No | High | Yes |
| 7620 | A | Int. | 1 | Fair | Fair | Low | Low | Low | High | High | Low | No | Low | Yes |

As treated categories: A = four components of the intervention evaluated with good performance, B = Three components of the intervention evaluated with good performance, C = Less than three components of the intervention evaluated with good performance. * The intercultural broker deserted within the first year. ^a^ The initial traditional midwife died and was replaced by the apprentice or another traditional midwife. High indicates that the proportion of household in the community is higher than the average, otherwise is Low.
