# Supplementary material for: Safe birth in cultural safety in southern Mexico: a pragmatic non-inferiority cluster-randomised controlled trial
Source: BMC Pregnancy Childbirth. 2022 Jan 17;22:43. doi: 10.1186/s12884-021-04344-w (PMC8762841; doi:10.1186/s12884-021-04344-w)
Supplement: Supplementary file 2 — Additional file 2. Comparison of groups as treated using GLMMs. The table shows the comparison of groups as treat using OR and 95% confidence intervals. [file 12884_2021_4344_MOESM2_ESM.docx]

**Supplementary material 2.**

**Comparison of groups as treated using GLMMs.**

**Table 1. OR and 95%CI for each comparison of groups as treated. We present in bold the measures showing significant differences.**

|  | **Four components**  **Good** | **Three components**  **Fear** | **Less than three components**  **Poor** | **Control** |
| --- | --- | --- | --- | --- |
| **Four components** |  | Childbirth complications  0.66 0.13–3.37  Neonatal complication  1.49 0.31–7.06  Perinatal death  0.71 0.09–5.37 | Childbirth complications  0.36 0.10–1.29  Neonatal complication  0.86 0.30–2.52  Perinatal death  1.22 0.27–5.56 | **Childbirth complications**  **0.35 0.14–0.92**  Neonatal complication  0.61 0.25–1.71  Perinatal death  0.66 0.25– 1.77 |
|  |  |  | Childbirth complications  0.32 0.10–1.06  Neonatal complication  0.68 0.25–1.81  Perinatal death  0.85 0.21–3.43 | |
|  |  | Childbirth complications  0.42 0.12–1.46  Neonatal complication  0.99 0.35–2.81  Perinatal death  1.02 0.22–4.66 | |  |
|  |  | Childbirth complications  0.34 0.11–1.12  Neonatal complication  0.72 0.27–1.94  Perinatal death  0.83 0.21–3.38 | | |
| **Three components** |  |  | Childbirth complications  0.54 0.15–1.96  Neonatal complication  0.60 0.14–2.20  Perinatal death  1.83 0.33–9.90 | Childbirth complications  0.47 0.14–1 .57  Neonatal complication  0.43 0.11–1.70  Perinatal death  1.05 0.21–5.09 |
| **Less than three components** | Childbirth complications  0.43 0.16–1.16  Neonatal complication  0.75 0.29–1.95  Perinatal death  1.49 0.37–6.06 | |  | Childbirth complications  0.87 0.45 – 1.68  Neonatal complication  0.72 0.32–1.64  Perinatal death  0.58 0.16–2.06 |
| **Control** | **Childbirth complications**  **0.37 0.15 –0.90**  Neonatal complication  0.54 0.23–1.29  Perinatal death  0.86 0.27–2.78 | |  |  |
| **Less than three components +**  **Control** | **Childbirth complications**  **0.39 0.16–0.93**  Neonatal complication  0.59 0.26– 1.36  Perinatal death  0.99 0.33–2.98 | |  |  |
